# Complete genome sequencing of four marine bacteria, *Gilvimarinus japonicus* NBRC 111987^T^, *Halioxenophilus aromaticivorans* JCM 19134^T^, *Maricurvus nonylphenolicus* JCM 17778^T^, and *Simiduia litorea* JCM 19759^T^ belonging to the family *Cellvibrionaceae*

**DOI:** 10.1128/mra.00873-24

**Published:** 2024-10-14

**Authors:** Takamasa Miura, Tomoyo Miyakawa, Yoko Kusuya, Kei Kamino

**Affiliations:** 1 Biological Resource Center, National Institute of Technology and Evaluation (NBRC), Chiba, Japan; University of Southern California, Los Angeles, California, USA

**Keywords:** *Cellvibrionaceae*, *Gilvimarinus japonicus*, *Halioxenophilus aromaticivorans*, *Maricurvus nonylphenolicus*, *Simiduia litorea*, PHB depolymerase

## Abstract

We report on the complete genomes of *Gilvimarinus japonicus* NBRC 111987^T^, *Halioxenophilus aromaticivorans* JCM 19134^T^, *Maricurvus nonylphenolicus* JCM 17778^T^, and *Simiduia litorea* JCM 19759^T^, isolated from the sea. Strains JCM 19134^T^, JCM 17778^T^, and JCM 19759^T^ contain genes predicted to be polyhydroxyalkanoate-degrading enzymes.

## ANNOUNCEMENT

The marine bacteria *Gilvimarinus* (1–5), *Halioxenophilus* (6), *Maricurvus* (7), and *Simiduia* (8, 9), which belong to the family *Cellvibrionaceae*, are known to degrade agar, cellulose, and organic compounds (some alkylphenols and xylenes). Genome sequencing of species belonging to this family will provide important information on the mode of degradation of various compounds in the sea.

The four strains were obtained from the Japan Collection of Microorganisms (JCM) and NITE Biological Resource Center (NBRC) and were cultured on Marine Agar 2216 (BD, New Jersey, USA) or NBRC medium 868 plates at 25°C for 2.5 days. Multiple colonies were scraped and genomic DNAs for both Illumina and Oxford Nanopore Technologies (ONT) libraries were purified using the MagAttract HMW DNA kit (Qiagen, Hilden, Germany). For Illumina sequencing, libraries were constructed using the QIAseq FX DNA Library Kit (Qiagen) and sequenced on an MiSeq instrument using the MiSeq Reagent Kit v3 (2 × 300 bp reads; Illumina, California, USA). For sequencing at ONT, libraries were prepared using the Native Barcoding Kit 24 V14 (SQK-NBD114.24; ONT, Oxford, UK) according to the manufacturer's instructions. To enrich for DNA fragments of >3 kb, we used Long Fragment Buffer (LFB) in the clean-up step after adapter ligation. Sequencing was performed using an R10.4.1 flow cell (FLO-MIN114; ONT) in combination with the MinION Mk1C platform (ONT). We applied data processing parameters to generate the FASTQ files, including high-accuracy model of base-calling with the minimum read length (1,000 bp), adapter trimming, and barcode trimming by using Guppy (v6.5.7) in MinKNOW (v23.4.5) software (https://community.nanoporetech.com/). Paired-end raw reads from the MiSeq sequencing were trimmed using fastp v0.23.2 software (10) with default parameters. These reads were split up using the split command in Seqkit v2.2 software (11) with option [-p 3 (-p four for NBRC 111987^T^)] and one of the read files was used for subsequent hybrid assembly. Long raw reads from ONT sequencing were trimmed using Nanofilt v2.8 software (12) with options (-q 8, -l 500, --headcrop 75). The hybrid assembly of the Illumina and ONT reads was performed using Unicycler v0.5 with default parameters (13). The DDBJ Fast Annotation and Submission Tool (DFAST) (https://dfast.ddbj.nig.ac.jp/) (14) and InterPro 99.0 (https://www.ebi.ac.uk/interpro/) were used for genome annotation with default parameters. The plasmid was determined by mlplasmids web server with default parameters (https://sarredondo.shinyapps.io/mlplasmids/) (15). The genome information used in the study is shown in Table 1.

**TABLE 1:** Genomic features of the four strains belonging to the family *Cellvibrionaceae*

Strain	*Gilvimarinus japonicus* NBRC 111987^T^	*Halioxenophilus aromaticivorans* JCM 19134^T^	*Maricurvus nonylphenolicus* JCM 17778^T^	*Simiduia litorea* JCM 19759^T^
Genome Size (bp)	4,195,725	5,942,233	5,859,353	4,429,217
Status	complete	complete	complete	complete
Genome Coverage (x)	104	89	239	187
No. of Sequences	1	1	2	1
*N_50_* Value (bp)	4,195,725	5,942,233	5,624,926	4,429,217
GC Content (%)	52.6	50.7	49.2	49.6
No. of CDSs	3,578	5,050	5,134	3,970
No. of rRNA Operons	3	3	3	3
Genome Accession No.	AP031500	AP031496	AP031498 (chromosome) AP031499 (pKU41E)	AP031497
DRA Accession No. (Illumina)	DRR546331	DRR546325	DRR546329	DRR546327
DRA Accession No. (ONT)	DRR546332	DRR546326	DRR546330	DRR546328
No. of reads (Illumina)	3,461,128	4,099,528	2,298,900	2,683,024
No. of reads (ONT)	58,614 (*N_50_* = 12,676)	114,117 (*N_50_* = 6,586)	353,519 (*N_50_* = 5,177)	165,032 (*N_50_* = 6,913)
Origin (Reference)	(3)	(6)	(7)	(9)

The genomes of JCM 19134^T^, JCM 17778^T^, and JCM 19759^T^ encoded polyhydroxyalkanoate (PHA) depolymerase and PHB depolymerase, known PHA degrading enzymes (16), containing a secretion signal. On the other hand, the genome of NBRC 111987^T^ did not encode these genes. Recently, *Halioxenophilus aromaticivorans* strain 4NH20-0024, the same strain as JCM 17778^T^, was reported to degrade the biodegradable plastics poly(3-hydroxybutyrate-co-3-hydroxyhexanoate) (PHBH) (17). The results suggest that three strains, JCM 19134^T^, JCM 17778^T^, and JCM 19759^T^, may be capable of biodegradation of PHA-based polymers outside cells.

## Data Availability

The complete genomes of *Gilvimarinus japonicus* NBRC 111987^T^, *Halioxenophilus aromaticivorans* JCM 19134^T^, *Maricurvus nonylphenolicus* JCM 17778^T^, and *Simiduia litorea* JCM 19759^T^ are available at DDBJ/ENA/GenBank under BioProject accession PRJDB17481. Genome accessions are available in Table 1.
